# Effects of Insulin-like Growth Factor 1 on the Maintenance of Cell Viability and Osteogenic Differentiation of Gingiva-Derived Mesenchymal Stem Cell Spheroids

**DOI:** 10.3390/medicina61010076

**Published:** 2025-01-04

**Authors:** Somyeong Hwa, Hyun-Jin Lee, Youngkyung Ko, Jun-Beom Park

**Affiliations:** 1 Department of Periodontics, College of Medicine, The Catholic University of Korea, Seoul 06591, Republic of Korea; somyeong.hwa@gmail.com (S.H.); hyunjinlee0423@gmail.com (H.-J.L.); ko_y@catholic.ac.kr (Y.K.); 2 Dental Implantology, Graduate School of Clinical Dental Science, The Catholic University of Korea, Seoul 06591, Republic of Korea; 3 Department of Medicine, Graduate School, The Catholic University of Korea, Seoul 06591, Republic of Korea

**Keywords:** cell survival, cell differentiation, cellular spheroids, insulin-like growth factor I, osteogenesis, stem cells

## Abstract

*Background and Objectives:* Insulin-like growth factor-1 (IGF-1) plays a vital role in various cellular processes, including those involving stem cells. This study evaluated the effects of IGF-1 on cell survival, osteogenic differentiation, and mRNA expression in gingiva-derived mesenchymal stem cell spheroids. *Materials and Methods:* Using concave microwells, spheroids were generated in the presence of IGF-1 at concentrations of 0, 10, and 100 ng/mL. Cellular vitality was qualitatively assessed using microscopy, while a water-soluble tetrazolium salt–based assay kit quantified cellular viability. Osteogenic differentiation was evaluated via alkaline phosphatase activity and an anthraquinone dye test to measure calcium deposition. Additionally, quantitative polymerase chain reaction (qPCR) analysis was performed to determine the expression of RUNX2 and COL1A1. *Results:* By day 1, the stem cell spheroids had successfully formed, and their morphology remained stable over the following 7 days. The IGF-1 concentrations tested showed no significant differences in cell viability. Similarly, alkaline phosphatase activity on day 7 revealed no observable changes. However, on day 7, the incorporation of IGF-1 led to an increase in Alizarin Red staining, indicative of enhanced calcium deposition. Notably, an IGF-1 concentration of 100 ng/mL significantly upregulated the expression of COL1A1. *Conclusions:* These findings suggest that IGF-1 supports the maintenance of cell viability and promotes the expression of COL1A1 in gingiva-derived mesenchymal stem cell spheroids, highlighting its potential role in enhancing osteogenic differentiation. Future research should include long-term studies to evaluate the sustainability of IGF-1-induced effects on stem cell spheroids.

## 1. Introduction

Insulin-like growth factor I (IGF-1), produced by osteoblasts and chondrocytes, plays an essential role in skeletal development and serves as a critical regulator of tissue growth and development in response to growth hormone stimulation [1,2]. The spatiotemporal release of insulin is a promising approach, as IGF-1 is widely acknowledged to induce both angiogenesis and osteogenesis [3]. IGF-1 and related growth factors play a pivotal role in regulating bone remodeling, healing, and growth [4]. Prior research has demonstrated a connection between bone development and osteogenesis mediated by IGF-1 [5]. For instance, in a sheep model, encapsulation of IGF-1 in microspheres facilitated improved bone growth [6]. Recent findings also indicate that osteocytes produce and release IGF-1, highlighting its role in regulating osteoblast and osteoclast activity as well as bone health through remodeling processes [7].

Additionally, alpha granules in platelet-rich plasma are known to contain IGF-1 and other cytokines [8]. Osteocytes, through the secretion of IGF-1, significantly influence bone and mineral metabolism [9]. Oral administration of bioencapsulated human IGF-1 has been shown to accelerate musculoskeletal cell growth, differentiation, and repair in diabetic fractures by enhancing bone volume, density, and area [10]. Furthermore, high IGF-1 expression has been associated with successful outcomes in masquelet therapy [11], and poly(lactic-co-glycolic acid) scaffolds loaded with IGF-1 have demonstrated efficacy in treating growth plate damage [12]. IGF-1 has also been proposed as a therapeutic agent for bone repair in chronic inflammatory conditions [13].

In recent years, stem cells have attracted significant attention in research and medicine [14,15,16]. These cells possess the ability to replenish or repair damaged tissues and organs [17] and are used to develop models for drug testing and disease research [18,19]. The combination of stem cells and growth factors has been reported to enhance bone regeneration and angiogenesis [20,21]. For example, canine mesenchymal stem cells require agonists such as IGF-1, osteoinductive protein NELL-1, or bone morphogenetic protein 2 (BMP-2) to achieve robust osteogenic development in vitro [2,22]. The application of nanoparticles loaded with dexamethasone enhanced the differentiation potential of human bone marrow stem cells [23]. Compared to bone morphogenetic protein 7, IGF-1 has shown superior capacity to promote osteogenic differentiation in mesenchymal stem cells [24]. The clinical significance of this study lies in IGF-1′s potential to regulate bone healing and remodeling, influence stem cell differentiation, and enhance osteoblast function. Our study specifically aimed to investigate the effects of IGF-1 on stem cell spheroids, focusing on their ability to maintain shape, enhance cellular vitality, stimulate osteogenic differentiation, and alter mRNA expression levels.

## 2. Materials and Methods

### 2.1. Cell Spheroids of Gingiva-Derived Mesenchymal Stem Cells

The study protocols, identified as KC21SISE0515 and KC24SISI0024, were reviewed and approved by the Institutional Review Board of Seoul St. Mary’s Hospital, College of Medicine, The Catholic University of Korea, on 3 August 2021 and 16 January 2024, respectively. Gingival tissue was obtained from a 37-year-old male undergoing gingivectomy to isolate mesenchymal stem cells, following previously established methods for isolation and characterization [25]. The cells were cultured in a dish, with non-adherent cells removed, and the culture medium was refreshed every two to three days.

To generate stem cell spheroids, concave microwells (H389600, StemFIT 3D; MicroFIT, Seongnam, Republic of Korea) were employed. Approximately 1 million stem cells were seeded into these microwells, facilitating cell–cell interactions. The design of the microwells promotes cellular adhesion, leading to the formation of uniform spheroids. Insulin-like growth factor-1 (IGF-1) was administered to the gingiva-derived mesenchymal stem cell spheroids at concentrations of 0, 10, and 100 ng/mL. This study included three groups of spheroids: Group 1 served as the control without IGF-1, Group 2 was treated with IGF-1 at 10 ng/mL, and Group 3 was treated with IGF-1 at 100 ng/mL. Morphological characteristics of the spheroids were assessed at various time points: days 1, 3, 5, and 7.

### 2.2. Assessment of Cellular Viability

During the cultivation of stem cells in osteogenic media, spheroid formation was observed. To assess the condition of the stem cell spheroids, we performed evaluations on days 3 and 7 using a dual-color assay that detects plasma membrane integrity and esterase activity. This assay utilized propidium iodide and the acetoxymethyl ester of calcein (Live/Dead Kit assay; Molecular Probes, Eugene, OR, USA) [26]. Additionally, cellular viability was quantitatively measured on days 1, 3, 5, and 7 using a water-soluble tetrazolium salt-based assay kit (Cell Counting Kit-8; Dojindo, Tokyo, Japan) [27].

### 2.3. Levels of Alkaline Phosphatase Activity and Calcium Deposition

Osteogenic differentiation was assessed by measuring alkaline phosphatase (ALP) activity and calcium deposition using an anthraquinone dye assay [28]. On day 7, cell spheroids cultured in osteogenic medium were harvested for evaluation. ALP activity was measured using a commercial kit (K412-500, BioVision, Inc., Milpitas, CA, USA). Calcium deposition, indicative of osteogenic differentiation, was evaluated on days 7 and 14 using an anthraquinone dye assay [29]. For this process, cell spheroids were removed from the culture wells, fixed, and washed. Alizarin Red S dye was applied to the samples at room temperature for 30 min to stain calcium deposits. The dye was then extracted using cetylpyridinium chloride for quantification.

### 2.4. Total RNA Extraction and Real-Time Polymerase Chain Reaction Quantification of RUNX2 and COL1A1

The quantity and quality of RNA were analyzed by measuring absorbance at 260 and 280 nm using an ND-2000 spectrophotometer (Thermo Fisher Scientific, Inc., Waltham, MA, USA). The expression levels of mRNA were determined using quantitative polymerase chain reaction (qPCR) amplification [30]. Primers were designed based on sequences available in GenBank, with the following forward and reverse sequences:

RUNX2: Forward 5′-AAT GAT GGT GTT GAC GCT GA-3′; Reverse 5′-TTG ATA CGT GTG GGA TGT GG-3′
COL1A1: Forward 5′-CCAGAAGAACTGGTACATCAGCAA-3′; Reverse 5′-TGGTTTCTTCTCCTCTGCGC-3′
β-actin: Forward 5′-TGGCACCCAGCACAATGAA-3′; Reverse 5′-CTAAGTCATAGTCCGCCTAGAAGC-3′

β-actin was used as the housekeeping gene to normalize the expression levels of RUNX2 and COL1A1.

### 2.5. Statistical Analysis

For each experiment, the mean and standard deviation were calculated. Normality and homogeneity of variance tests were conducted to verify that the data followed a normal distribution and exhibited equal variances. A two-way analysis of variance (ANOVA) was used to analyze the effects of IGF-1 concentration and time points, while a one-way ANOVA was employed to assess differences among groups. Tukey’s post hoc test was applied for pairwise comparisons (SPSS 12 for Windows; SPSS Inc., Chicago, IL, USA). All experiments were performed in triplicate. Statistical significance was determined at a *p*-value of <0.05.

## 3. Results

### 3.1. Generation of Spheroid-Shaped Stem Cell Aggregates

**Figure 1:**
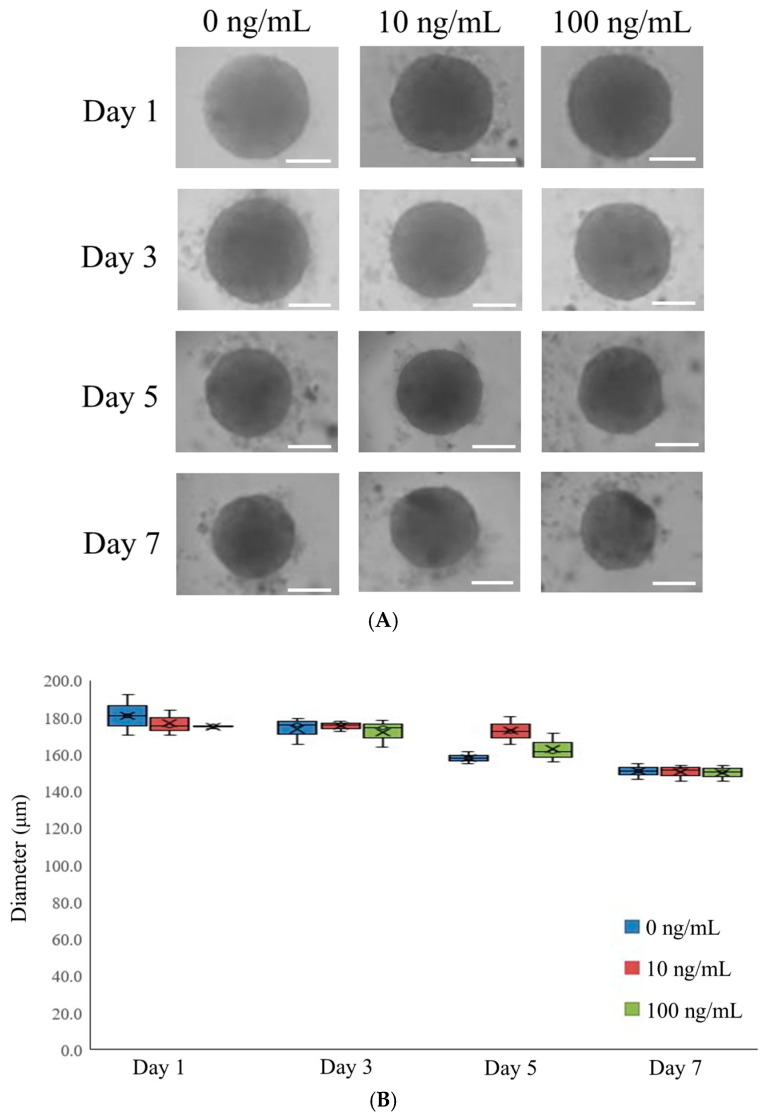
Morphological evaluation. (**A**) The morphology of stem cell spheroids at days 1, 3, 5, and 7. The scale bar represents 100 μm. (**B**) The diameters of the stem cell spheroids on days 1, 3, 5, and 7. The figure is a graphical representation, with x signifying the mean value, and the upper and lower limits denoting the maximum and minimum values, respectively. The box contains three lines, which represent the upper and lower quartiles and the median, respectively.

The morphological changes in stem cell spheroids treated with varying IGF-1 concentrations (0, 10, and 100 ng/mL) were assessed on days 1, 3, 5, and 7 (Figure 1A). Over the seven-day period, no significant alterations in spheroid morphology were observed. Although minor differences in spheroid size were noted between days 1 and 7, the spheroids consistently maintained their rounded shape. The diameters of the spheroids for each time point are presented in Figure 1B. On day 1, the spheroid diameters for the 0, 10, and 100 ng/mL IGF-1 groups were 179.9 ± 10.8 µm, 175.5 ± 6.8 µm, and 173.8 ± 0.9 µm, respectively, with no statistically significant differences between groups (*p* > 0.05). Similar results were observed on days 3, 5, and 7 (*p* > 0.05).

### 3.2. Assessment of Cell Viability

**Figure 2:**
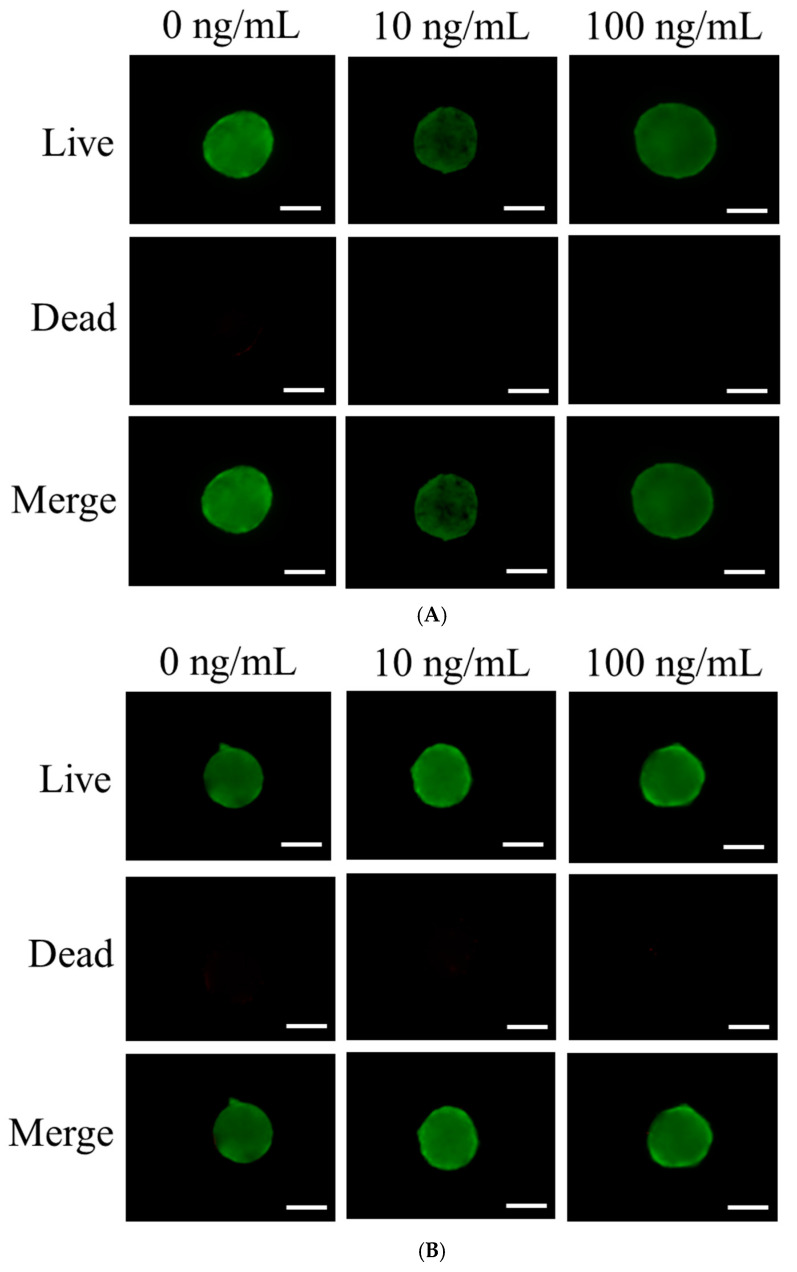

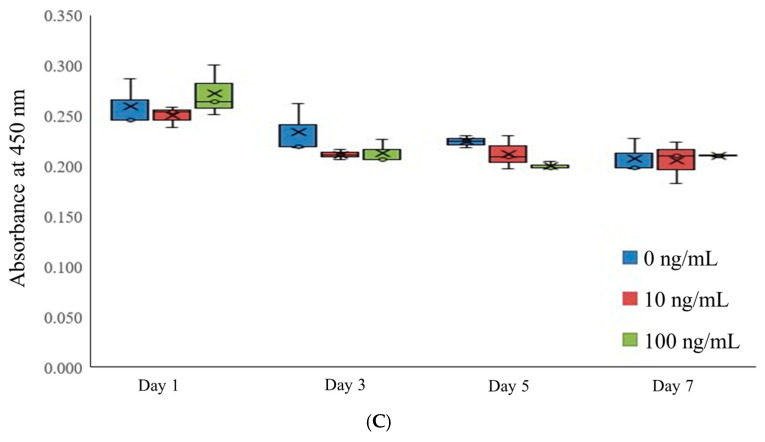
Composite images depicting live and dead stem cell spheroids. (**A**) Composite images of live, dead, and merged views of stem cell spheroids on day 3. The scale bar represents 100 μm. (**B**) Composite images of live, dead, and merged images of stem cell spheroids on day 7. (**C**) Quantification of cellular viability using a CCK-8 assay on days 1, 3, 5, and 7. The graph is a representation of data with the mean value. The upper and lower limits depict the maximum and minimum values, respectively, while the box encompasses three lines, which signify the upper and lower quartiles and the median, respectively.

Stem cell viability was qualitatively assessed on days 3 and 7 using a live/dead kit assay (Figure 2A,B). On day 3, most cells exhibited round morphologies with vibrant green fluorescence, indicating viability (Figure 2A). By day 7, green fluorescence persisted, demonstrating sustained cell viability over the extended incubation period (Figure 2B).

Quantitative assessments of cellular viability were conducted on days 1, 3, 5, and 7 using a water-soluble tetrazolium salt-based assay (Figure 2C). On day 1, the absorbance values at 450 nm for the 0, 10, and 100 ng/mL IGF-1 groups were 0.260 ± 0.024, 0.251 ± 0.010, and 0.272 ± 0.026, respectively, with no significant differences (*p* > 0.05). Similarly, on day 3, the respective values were 0.234 ± 0.025, 0.212 ± 0.005, and 0.214 ± 0.012 (*p* > 0.05). On day 5, the values for the same groups were 0.225 ± 0.006, 0.213 ± 0.017, and 0.201 ± 0.004 (*p* > 0.05). By day 7, the absorbance values were 0.208 ± 0.017, 0.206 ± 0.021, and 0.211 ± 0.001, again with no significant differences between groups (*p* > 0.05).

### 3.3. Alkaline Phosphatase Activity Levels and Extent of Calcium Deposition

**Figure 3:**
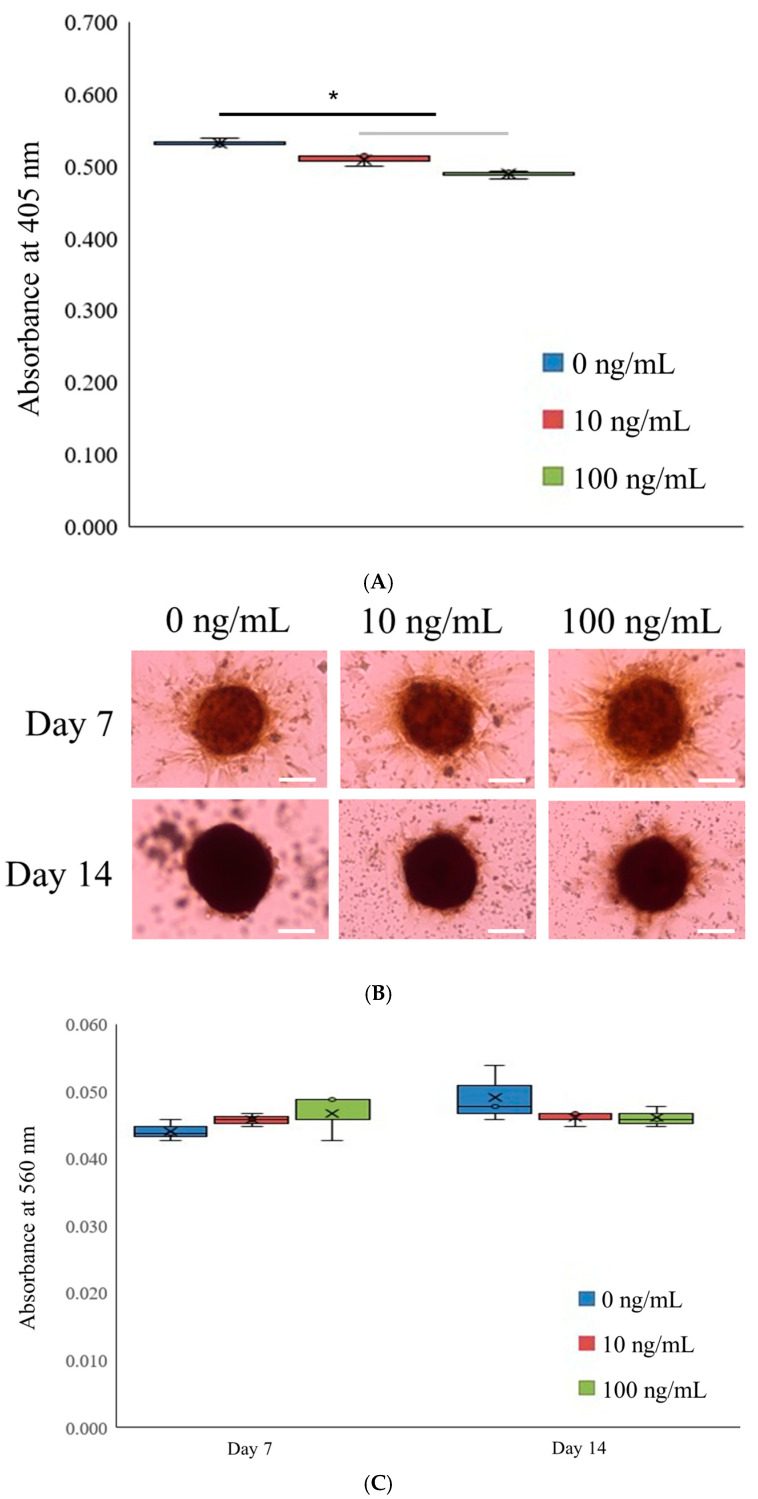
Osteogenic maturation of stem cell spheroids. (**A**) Alkaline phosphatase activity levels on day 7. * *p* < 0.05 compared to the 0 ng/mL group on day 7. (**B**) Microscopic assessment of outcomes from Alizarin Red S staining on days 7 and 14. A 100 μm scale bar is included. (**C**) Quantitative evaluation of Alizarin Red S staining. The figure features a graph that visualizes the data with the central tendency, denoted by the letter x, signifying the mean. The upper and lower boundaries represent the extremes of the data, showcasing the maximum and minimum values, respectively. The box highlights three lines, illustrating the upper and lower quartiles and the median, respectively.

The results of the alkaline phosphatase (ALP) activity assessment on day 7 are presented in Figure 3A. The absorbance values at 405 nm for the 0, 10, and 100 ng/mL IGF-1 groups were 0.532 ± 0.005, 0.509 ± 0.009, and 0.488 ± 0.006, respectively (*p* < 0.05).

Calcium deposition, as measured using Alizarin Red staining, was evident across all groups on both days 7 and 14 (Figure 3B). On day 7, the absorbance values at 405 nm were 0.044 ± 0.002, 0.046 ± 0.001, and 0.047 ± 0.003 for the 0, 10, and 100 ng/mL IGF-1 groups, respectively (*p* > 0.05) (Figure 3C). On day 14, the corresponding absorbance values were 0.049 ± 0.004, 0.046 ± 0.001, and 0.046 ± 0.002 (*p* > 0.05).

### 3.4. Quantitative Real-Time Polymerase Chain Reactions of RUNX2 and COL1A1

**Figure 4:**
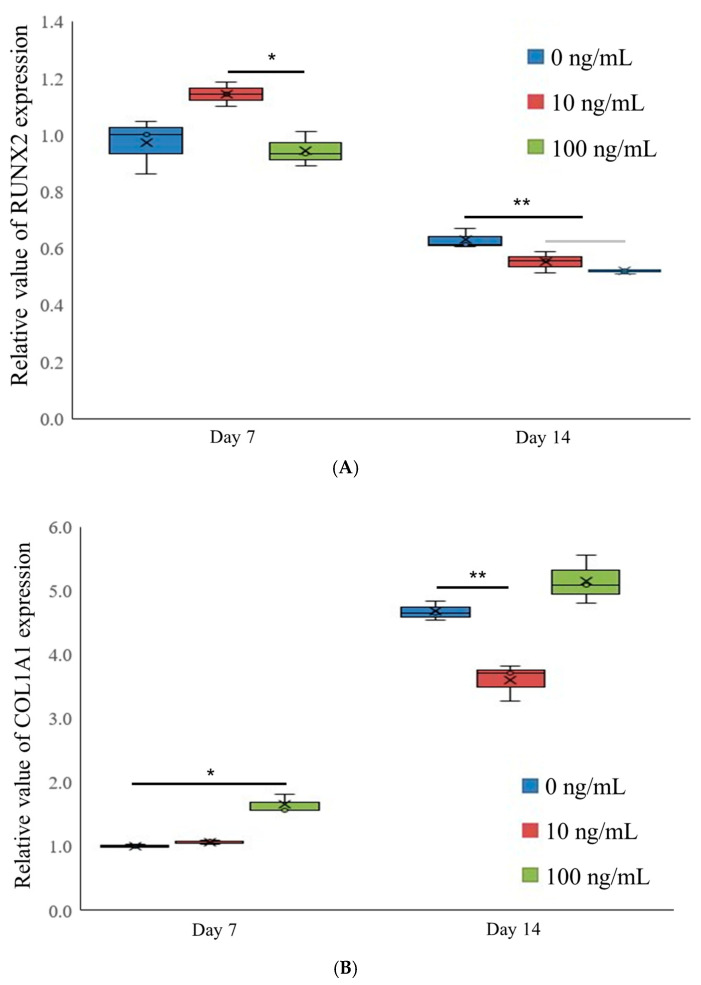
Quantification by real-time polymerase chain reaction on day 7. (**A**) Expression of RUNX2. * *p* < 0.05 compared to the 10 ng/mL group on day 7. ** *p* < 0.05 compared to the 0 ng/mL group on day 14. (**B**) Expression of COL1A1. * *p* < 0.05 compared to the 0 ng/mL group on day 7. ** *p* < 0.05 compared to the 0 ng/mL group on day 14. The graph visually represents the central tendency, denoted by the letter x, which signifies the mean value. The upper and lower boundaries of the graph represent the extremes of the data, showcasing the maximum and minimum values, respectively. The box has three lines, illustrating the upper and lower quartiles and the median, respectively.

mRNA expression levels of RUNX2 and COL1A1 were analyzed using qPCR (Figure 4A,B). On day 7, the mRNA expression of RUNX2 in the 0, 10, and 100 ng/mL IGF-1 groups was 1.000 ± 0.100, 1.178 ± 0.044, and 0.974 ± 0.064, respectively (*p* < 0.05). On day 14, RUNX2 expression levels decreased across all groups, with values of 0.648 ± 0.036, 0.569 ± 0.038, and 0.534 ± 0.008, respectively.

In contrast, COL1A1 mRNA expression showed significant variation. On day 7, the expression levels were 1.000 ± 0.029, 1.058 ± 0.028, and 1.625 ± 0.140 for the 0, 10, and 100 ng/mL groups, respectively, with the 100 ng/mL group showing a significant increase (*p* < 0.05). By day 14, the respective expression levels of COL1A1 were 4.555 ± 0.145, 3.515 ± 0.276, and 5.018 ± 0.364, indicating sustained upregulation, particularly in the 100 ng/mL IGF-1 group.

## 4. Discussion

This study investigated the effects of IGF-1 on stem cell spheroids, specifically their ability to maintain morphology, enhance cellular vitality, and promote osteogenic differentiation. Previous studies have extensively explored the application of IGF-1 in various contexts [31,32,33]. For instance, an IGF-1–gelatin sponge complex was locally applied in a rat model of osteoporosis, resulting in enhanced osseointegration and increased bone tissue formation around implants [31]. Similarly, in a rabbit femoral defect model, the addition of IGF-1 in silicon- and zinc-doped brushite cement demonstrated improved bone repair [32]. However, localized administration of 4 μg of IGF-1 did not promote osseointegration two weeks post-surgery in either healthy rabbits or those with osteoporosis [33]. In this study, stem cells were utilized without the support of a scaffold, forming spheroids to evaluate the direct effects of IGF-1. While scaffolds were not employed here, numerous studies have demonstrated the effectiveness of various scaffold materials in facilitating stem cell applications. These include β-tricalcium phosphate, calcium phosphate, absorbable gelatin sponges, laminin gel, and poly(lactic-co-glycolic acid) (PLGA) membranes or microspheres [31,34,35,36,37,38,39]. These scaffolds provide structural support, enhance cell adhesion, and enable controlled delivery of bioactive molecules, such as IGF-1, which can synergistically promote bone regeneration and tissue engineering.

The dose of IGF-1 applied in previous studies has varied significantly depending on the experimental design and objectives [24,31,33,36,37,38,40,41]. For example, IGF-1 combined with transforming growth factor-β1 effectively induced the osteogenic potential of extraction socket tissue cells at concentrations up to 2.5 ng/mL [40]. In another study, IGF-1 was applied at concentrations of 400–800 ng/mL to enhance the osteogenic differentiation of bone marrow-derived stem cells [24]. Delivery systems have been developed to optimize IGF-1 release and efficacy. For instance, calcium phosphate cement was used to encapsulate IGF-1 and BMP-2 at a dose of 10 µg/g, enabling sequential release in an osteoblast model [36]. Absorbable collagen sponges impregnated with 13 μg of IGF-1 were successfully applied to repair mouse calvarial defects [38]. Similarly, absorbable gelatin sponge particles loaded with 10 μg of IGF-1 were used to enhance bone regeneration around dental implants in a rat model (30). In another study, microparticles were loaded with either 2 μg of IGF-1, 2 μg of BMP-2, or a combination of 1 μg of IGF-1 and BMP-2, and were injected into a defect site in a rat model, resulting in improved bone healing [41]. A 4 μg dose of IGF-1 was administered during ostectomy procedures prior to implant insertion in a rabbit model, though results varied depending on the context [33]. Additionally, when 10 μg of IGF-1 and 0.8 μg of vascular endothelial growth factor (VEGF) were incorporated into a PLGA membrane, periodontal outcomes improved, with decreased probing depth and increased clinical attachment levels [37]. The beneficial effects may be concentration-dependent, such that low doses may exhibit osteogenic properties, whereas increasing the concentration beyond a certain threshold could be detrimental to the cells [42]. In the present study, we investigated the effects of IGF-1 on the cellular viability of gingiva-derived mesenchymal stem cells within spheroids. We employed IGF-1 concentrations of 10 and 100 ng/mL, which are within the lower range compared to some previous studies. These concentrations did not result in significant changes in cellular viability. However, our findings demonstrated that IGF-1 at these doses enhanced spheroid functionality, particularly in terms of osteogenic differentiation markers.

The modes of action of IGF-1 encompass several pathways [43,44]. In response to thrombin stimulation, platelets exhibited no IGF-1 upregulation [43]. Osteoblast differentiation was promoted by CD301b+ macrophages through the involvement of IGF-1 [44]. Intriguingly, in osteoblasts lacking IGF-1 or its receptor, parathyroid hormones could not effectively stimulate bone formation [45]. The use of three-dimensional titanium scaffolds enhanced osteogenic differentiation and new bone formation by adipose tissue-derived stem cells, along with an increased expression of components within the IGF-1R/AKT/mTORC1 pathway. This was evident through increased expression of osteogenesis-related mRNAs and proteins, including IGF-1 [46,47]. Open-flap debridement with leukocyte–platelet-rich fibrin application significantly increased IGF-1 at 2 weeks compared with the baseline [48].

We explored the impact of IGF-1 on the capacity of stem cell spheroids to assess mRNA expression levels of RUNX2 and COL1A1 in osteogenic differentiation. This study demonstrated that IGF-1 administration had a tendency to increase RUNX2 expression and statistically increase COL1A1 expression, indicating a direct increase in osteogenesis. Moreover, the outcomes of this investigation indicated that administration of IGF-1 led to a significant increase in the expression of COL1A1. We selected RUNX2 and COL1A1 as the focus of qPCR analysis in stem cells exposed to IGF-1 because of their important roles in bone formation and remodeling [49]. IGF-1 is notable for its significant involvement in various cellular processes, including stem cell function, and is essential for proper bone development in the skeletal system. It operates as a vital regulator of tissue growth and development, responding to growth hormone stimulation. Previous research demonstrated IGF-1′s crucial role in controlling bone remodeling, healing, and growth, indicating its potential connection to bone development and osteogenesis. RUNX2 serves as a crucial transcription factor in regulating osteoblast differentiation, playing a vital role in skeletal development [50]. It directly activates several osteoblast-specific genes, including COL1A1, which encodes one of the two chains of type I collagen, the primary collagen in bone and a critical component of the extracellular matrix, providing strength and structure to bone tissue [51]. Expression of COL1A1 indicates collagen synthesis, which is essential for structural integrity and strength in bone tissue [51]. Examining the expression of RUNX2 and COL1A1 can provide valuable insights into the osteogenic potential and differentiation of stem cells in response to IGF-1 treatment.

IGF-1 actions have been synergistically improved by adding other substances [35,36,39,41,52,53,54]. Critical-sized bone defect repair in a rat model was increased by simultaneous infusion of IGF-1 and BMP-2, which then were sequentially released [41]. This release increased the osteogenic proliferation and differentiation of mouse osteoblasts [36]. A scaffold of β-tricalcium phosphate was coated with IGF-1 and BMP-2 to stimulate bone regeneration in vivo while maintaining biosafety [35]. Another study found that osteoblastic differentiation and proliferation of cementoblasts resulted from regulated and sequential introduction of IGF-1 and bone morphogenetic factor 6 (a progression factor) using chitosan/alginate/poly(lactic-co-glycolic acid) hybrid scaffolds [52]. Three-dimensional nanofiber membranes loaded with IGF-1 and BMP-2 were seeded with bone marrow-derived mesenchymal stem cells as a suitable barrier membrane, demonstrating both osteoinduction and osteoconduction [53]. The application of bone marrow-derived stem cells with IGF-1 and transforming growth factor β1 into a laminin gel scaffold enhanced the restoration of hyaline cartilage in osteochondral defects [39]. A previous study demonstrated that rabbits treated with a combination of IGF-1 and platelet-derived growth factor showed a higher positive effect on bone regeneration compared with a platelet-rich, plasma-treated group or controls [54].

Evaluating alkaline phosphatase activity is a key assay in bone biology research, particularly when examining the osteogenic differentiation potential of stem cells [55]. Alkaline phosphatase is an enzyme that is highly expressed in osteoblasts during the early stages of bone formation and is considered a hallmark of osteoblast differentiation and maturation [56]. As a result, it is a crucial marker for assessing the osteogenic capacity of stem cells in vitro, making it a vital tool in the field of bone biology research [57]. This assay is a cornerstone in the field of bone biology, providing a reliable and accessible method to assess osteogenic differentiation. Researchers can gain valuable insights into the mechanisms of bone formation and the potential of various treatments to enhance bone regeneration by evaluating alkaline phosphatase activity [58]. Alizarin Red S is a fluorescent dye that binds to calcium in tissues, resulting in red-colored pigmentation [59]. In this study, the application of IGF-1 demonstrated a trend of increased Alizarin Red S staining in the early period, though the difference was not statistically significant.

Enhancing the effects of IGF-1 could be achieved synergistically by incorporating other compounds [35,36,39,41,52,53,54]. In a rat model of critical-sized bone defects, co-administration of IGF-1 and bone morphogenetic protein-2 resulted in an augmented repair process. This strategy involved the sequential release of BMP-2 and IGF-1, ultimately leading to increased bone defect healing [41]. Mouse osteoblast proliferation and differentiation were positively influenced by the consecutive release of IGF-1 and bone morphogenetic protein-2, contributing to bone health [36]. A scaffold composed of β-tricalcium phosphate was coated with IGF-1 and bone morphogenetic protein-2 to promote in vivo bone regeneration while ensuring safety [35]. In another investigation, controlled and sequential introduction of IGF-1 as a competence factor and bone morphogenetic factor-6 as a progression factor via chitosan/alginate/poly (lactic-co-glycolic acid) hybrid scaffolds induced osteoblastic differentiation and proliferation of cementoblasts [52]. Utilizing three-dimensional nanofiber membranes loaded with IGF-1 and bone morphogenetic protein-2, and seeded with bone marrow-derived mesenchymal stem cells, an effective approach was devised that acted as a suitable barrier membrane with both osteoinductive and osteoconductive properties [53]. The incorporation of bone marrow-derived stem cells alongside IGF-1 and transforming growth factor-β1 into a laminin gel scaffold yielded improved restoration of hyaline cartilage in osteochondral defects [39]. Notably, a study in rabbits demonstrated that the combination of IGF-1 and platelet-derived growth factor produced more favorable outcomes for bone regeneration compared to platelet-rich plasma-treated groups or control groups in rabbit models [54]. Combined treatment of IGF-1 and electrical stimulation promoted bone regeneration in vitro using MC3T3-E1 cells [60]. IGF-1 and insulin have been increasingly acknowledged as inducible factors for osteogenesis and angiogenesis, and their local delivery has been utilized for bone regeneration [3,61].

There are limitations to consider when interpreting this study. The evidence that IGF-1 plays a role in the viability of gingiva-derived mesenchymal stem cell spheroids and enhances osteogenic differentiation is preliminary. More extensive experiments are required to confirm the effects of IGF-1 on cell viability, particularly over longer durations and under varying conditions. To fully understand the impact of IGF-1 on the multipotency of these stem cells, it is crucial to investigate other differentiation pathways, such as lipogenic differentiation. Evaluating the potential of IGF-1 to influence adipogenic, chondrogenic, or even myogenic differentiation would offer a more comprehensive understanding of its role in stem cell biology. Previous research into the mechanisms of bone repair highlighted the critical role and significance of stem cells [62]. A previous report suggests that application of osteo-organoid-derived mesenchymal stem cells offers a solution to the challenges posed by the limitations of autologous cells and the potential risks linked to allogeneic sources in stem cell transplantation [63]. A previous study highlighted the significance of biomaterials, noting that understanding cellular behaviors and the activation of signaling pathways can provide valuable insights when designing biomaterials with enhanced properties for bone tissue engineering [64]. A previous study demonstrated that bone-targeted lipid nanoparticles delivering RUNX2 mRNA significantly enhanced bone repair and formation in a bone defect model [65]. Stem cell technology is expected to play a pivotal role in the advancement of functional biomaterials, and additional studies are expected to contribute to this dynamic and promising field [66].

The clinical implications of our study are significant. IGF-1’s ability to promote osteogenic differentiation makes it a promising tool for improving outcomes in bone grafts, dental implants, and the healing of bone fractures, which are common in clinical settings. The enhanced expression of COL1A1, a gene crucial for the synthesis of collagen type I (the primary component of the bone matrix), suggests that IGF-1 treatment can improve the quality and strength of newly formed bone tissue to reduce recovery times and enhance patient outcomes. The dose-dependent effects of IGF-1 reported here also suggest that IGF-1 treatments could be tailored to individual patients based on the severity of bone damage or the specific requirements of bone tissue engineering applications. Such personalized treatment strategies could revolutionize the management of bone defects and orthopedic surgeries, offering more efficient and effective regeneration of bone tissue. Additional research is needed to address limitations and pave the way for potential clinical applications. Future research should include a wider range of concentrations, long-term evaluation, mechanism studies, and eventually clinical trials.

This investigation looked at how IGF-1 affects the ability of stem cell spheroids to maintain their morphology, increase their cellular vitality, and promote osteogenic differentiation. Overall, we demonstrated that the presence of IGF-1 preserved cell viability and improved COL1A1 expression in stem cell spheroids. To further develop this approach, future studies should explore advanced IGF-1 delivery systems, investigate synergistic effects with other osteogenic and angiogenic growth factors, and validate these findings in dynamic in vitro or in vivo models. This approach holds significant potential for advancing IGF-1-based therapies into clinical applications for bone tissue engineering and stem cell-based regenerative therapy.

## Data Availability

This article contains all of the information that was created or examined during this investigation.
